# A Case of Large Cell Neuroendocrine Carcinoma of the Bladder With Long‐Term Control by Metastasectomy

**DOI:** 10.1002/iju5.70102

**Published:** 2025-10-01

**Authors:** Naoto Takaoka, Kosuke Kobayashi, Takehiro Yamane, Kazuma Hiramatsu, Kazunari Tsuchihashi, Hiroaki Kawanishi, Hiroyasu Yamashiro, Tomitaka Nakayama, Kazuhiro Okumura

**Affiliations:** ^1^ Department of Urology Tenri Hospital Nara Japan; ^2^ Department of Breast Surgery Tenri Hospital Nara Japan; ^3^ Department of Orthopedic Surgery Tenri Hospital Nara Japan

**Keywords:** large cell neuroendocrine carcinoma, metastasectomy, partial cystectomy, subcutaneous metastasis, urinary bladder

## Abstract

**Introduction:**

Large cell neuroendocrine carcinoma (LCNEC) of the bladder is rare and aggressive. Reports of metastasectomy for single distant metastases are even rarer.

**Case Presentation:**

A 49‐year‐old man was admitted to our hospital with gross hematuria. Cystoscopy, magnetic resonance imaging, and computed tomography revealed invasive bladder cancer without metastasis. He underwent transurethral resection of the bladder tumor, and histopathological examination confirmed LCNEC. The patient received neoadjuvant chemotherapy followed by partial cystectomy. Nineteen months postoperatively, a solitary metastasis was detected in the right axillary lymph node, and at 44 months, another solitary metastasis appeared in the subcutaneous tissue outside the right scapula. Both metastases were surgically resected, and no additional treatment was administered. At the time of this writing, there had been no recurrence or metastasis for 42 months following the second metastasectomy.

**Conclusion:**

Metastasectomy may be an effective treatment option for solitary metastasis of LCNEC of the bladder.

**Abbreviations**
CT: computed tomography
FDG PET: fluorodeoxyglucose positron emission tomography
LCNEC: large cell neuroendocrine carcinoma

**Summary**

- If metastases of LCNEC of the bladder are solitary and resectable, metastasectomy may be a viable treatment option.

## 1 Introduction

Large cell neuroendocrine carcinoma (LCNEC) of the bladder is rare and aggressive, often presenting with metastasis or recurrence. The presence of metastasis is associated with poorer survival. We herein report a case in which a solitary metastasis of bladder LCNEC was detected twice, and long‐term disease control was achieved through repeated metastasectomy.

## 2 Case Presentation

**FIGURE 1:**
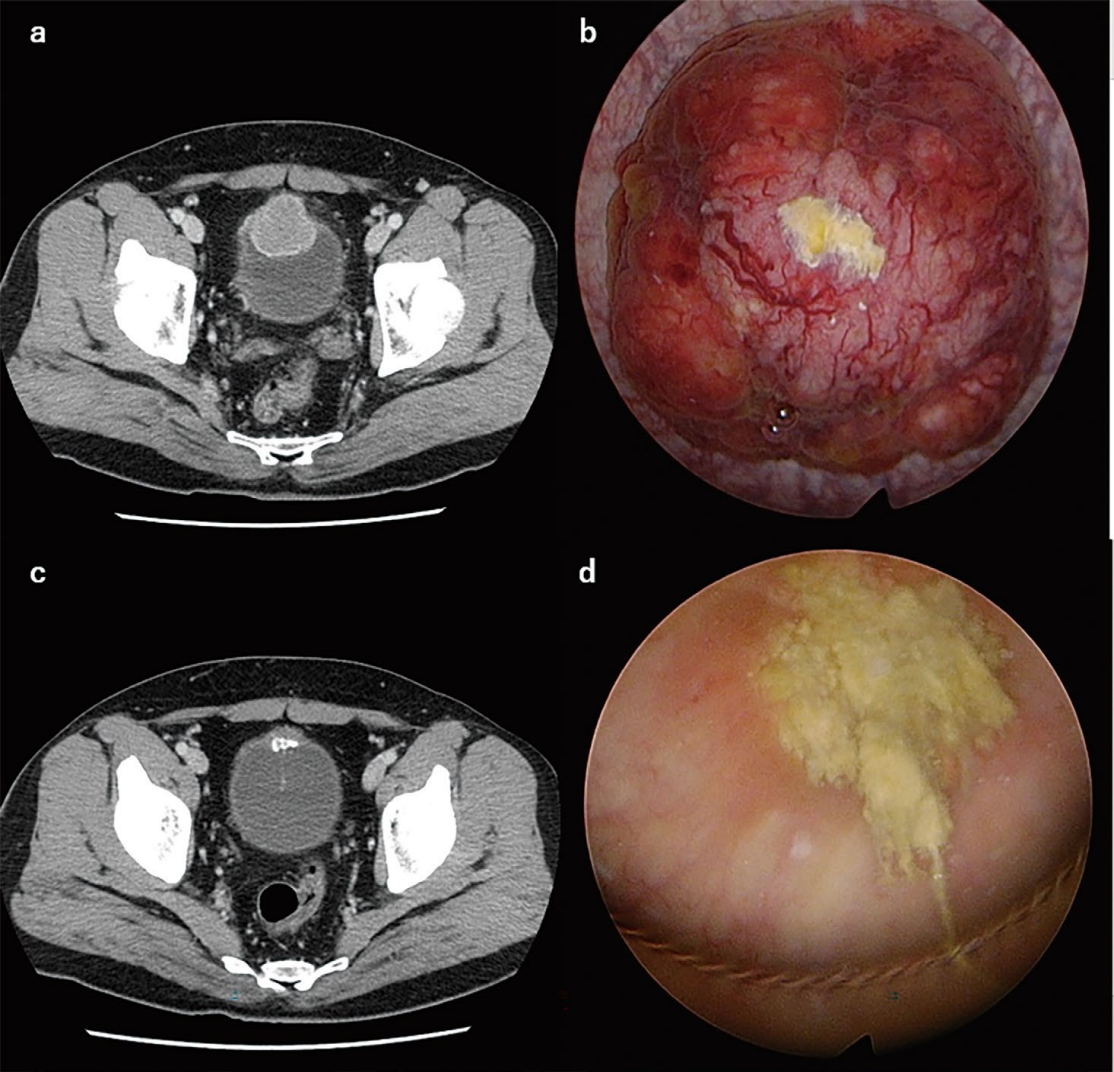
Pelvic computed tomography and cystoscopy images showing the bladder tumor (a, b) before transurethral resection and (c, d) after chemotherapy.

A 49‐year‐old man presented to our department with a 1‐month history of gross hematuria. He was a heavy smoker but had no other medical history and no family history of cancer. Urine cytology showed no evidence of malignancy. Cystoscopy, pelvic magnetic resonance imaging, and contrast‐enhanced computed tomography (CT) revealed a 36 × 45 × 45‐mm tumor located at the bladder dome, with invasion into the serosal layer (Figure 1a,b). Contrast‐enhanced CT of the brain, thorax, abdomen, and pelvis showed no signs of metastasis. The clinical diagnosis was cT3bN0M0.

**FIGURE 2:**
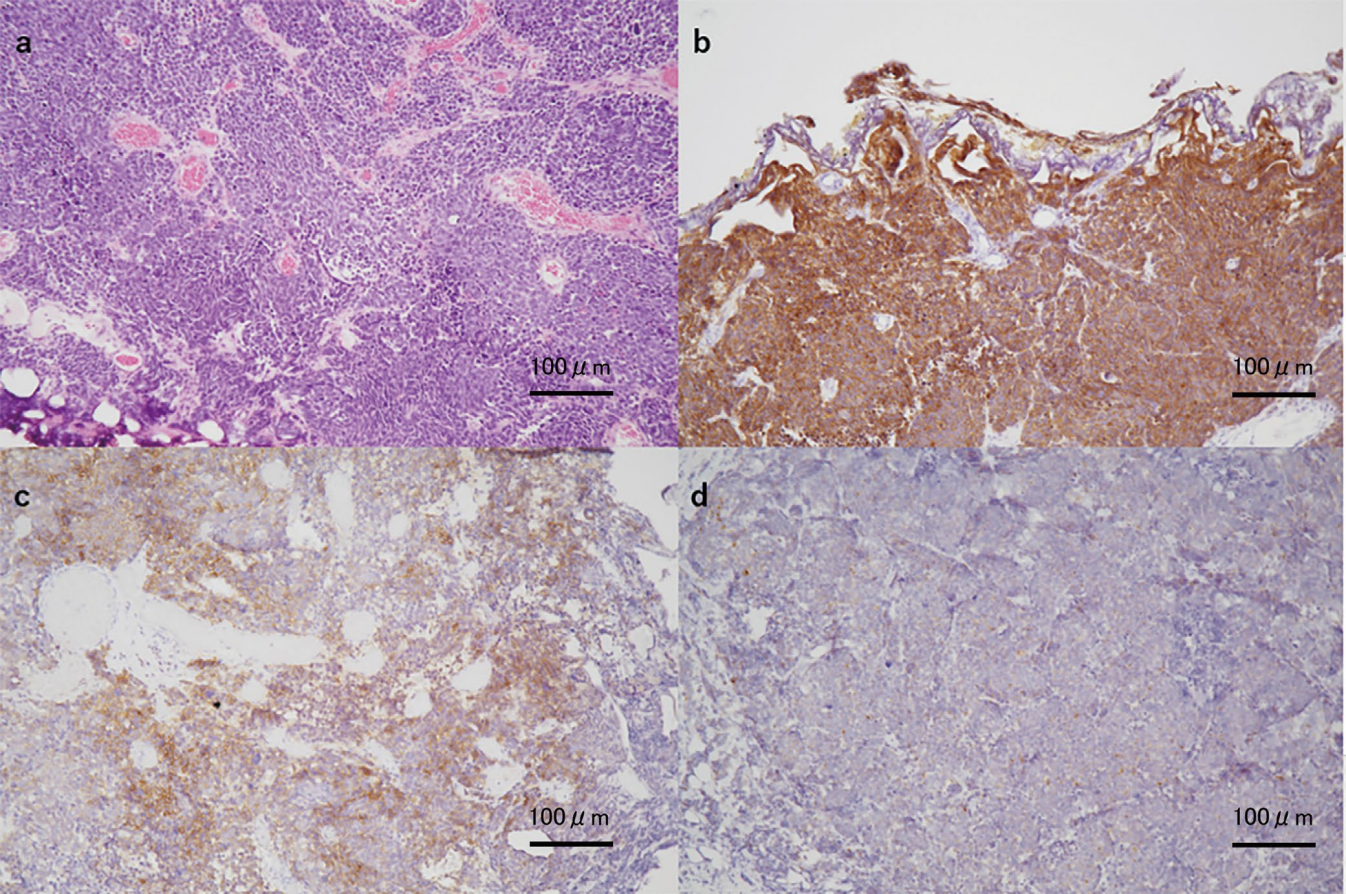
Pathological findings of the bladder tumor. Low‐magnification views (×100) showing (a) hematoxylin and eosin staining, (b) strong positivity for synaptophysin, (c) partial positivity for CD56, and (d) negativity for chromogranin A. CD56, cluster of differentiation 56.

The patient underwent transurethral resection of the bladder tumor. This surgery was performed only by sampling. Pathologic examination showed large cells with prominent nuclei infiltrating the muscle layer. Immunohistochemical staining revealed strong positivity for synaptophysin, partial positivity for cluster of differentiation 56, and negativity for chromogranin A (Figure 2). Overall, the histopathological findings were consistent with LCNEC.

The patient received two cycles of neoadjuvant chemotherapy with etoposide and cisplatin. CT after chemotherapy showed partial response (Figure 1c). The patient wished to preserve bladder function; therefore, because of the tumor location and the response to chemotherapy, we performed laparoscopic partial cystectomy without lymph node dissection. At the time of surgery, the tumor and surrounding tissues were visualized with cystoscopy, and the tumor was removed with sufficient margins (Figure 1d). Pathologic examination of the resected specimen showed no residual tumor, so the patient was followed up without adjuvant therapy.

**FIGURE 3:**
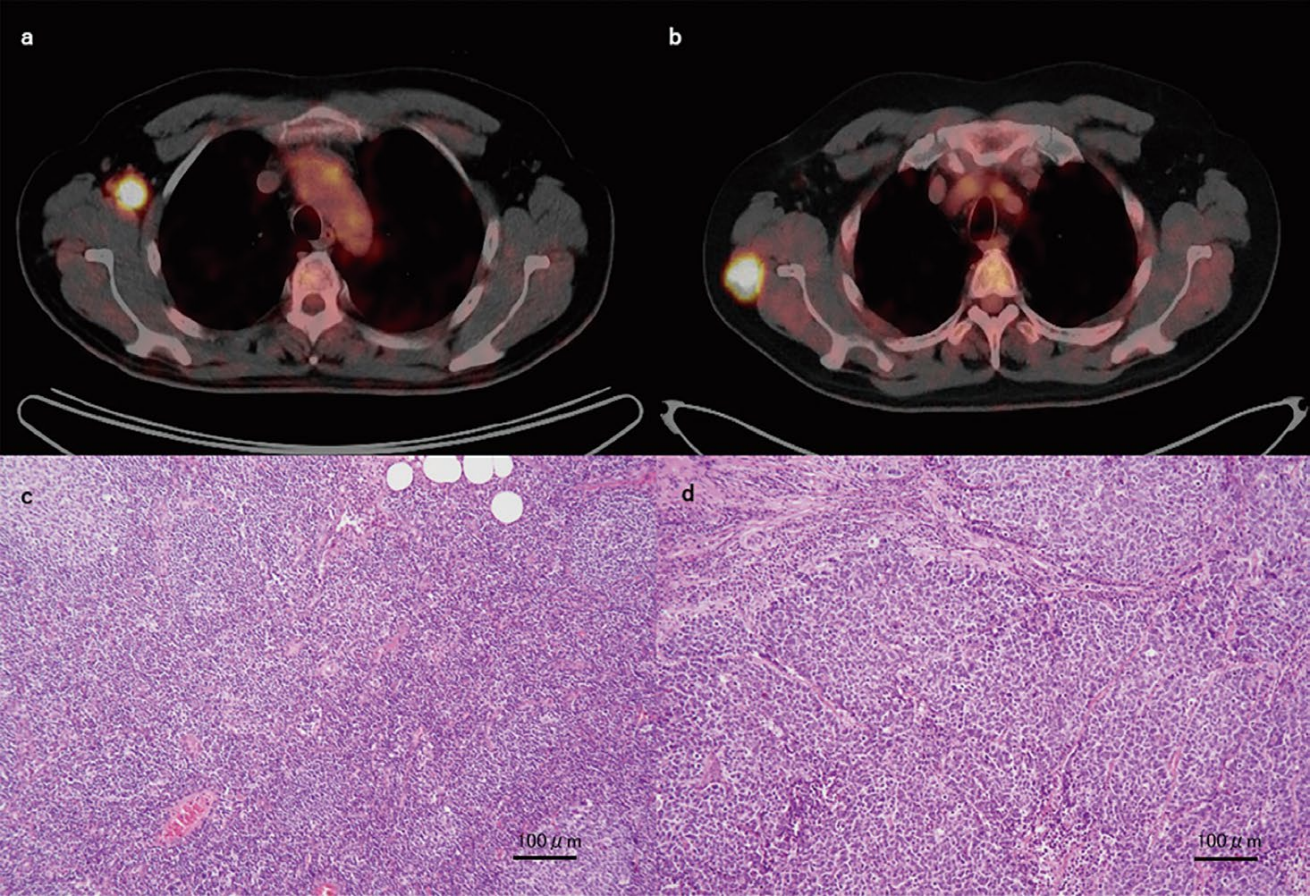
Fluorodeoxyglucose positron emission tomography findings. Fluorodeoxyglucose uptake is observed in (a) the right axillary lymph node and (b) the subcutaneous mass outside the right scapula. Microscopic findings of (c) the right axillary lymph node and (d) the subcutaneous mass outside the right scapula (hematoxylin and eosin staining, ×100).

Nineteen months after the partial cystectomy, CT revealed right axillary lymph node metastasis. Fluorodeoxyglucose (FDG) positron emission tomography (PET)/CT confirmed isolated right axillary lymph node involvement with a maximum standardized uptake value of 10.9 (Figure 3a). Because the metastasis was solitary and resectable, we decided to perform metastasectomy, which was performed by breast surgeons. Pathology confirmed LCNEC metastasis in the right axillary lymph node (Figure 3b). Because the metastasis had been completely excised, we opted for follow‐up without additional treatment.

Twenty‐five months after the metastasectomy, CT revealed a subcutaneous mass outside the right scapula. FDG PET confirmed the lesion as a subcutaneous metastasis, with a maximum standardized uptake value of 31.1, and no other metastases were suspected (Figure 3c). The patient underwent a second metastasectomy, performed by orthopedic surgeons. Pathologic analysis confirmed LCNEC metastasis (Figure 3d). As before, we continued with surveillance.

We followed the patient by performing cystoscopy and CT every 3 months postoperatively. At 42 months following the second metastasectomy, there was no evidence of recurrence or metastasis on CT or cystoscopy. In accordance with the patient's wishes, the follow‐up frequency was changed to every 6 months.

## 3 Discussion

Neuroendocrine carcinoma of the urinary bladder is classified into small cell neuroendocrine carcinoma, large cell neuroendocrine carcinoma, and paraganglioma [1]. According to data reported by Lopedote et al. [2], LCNEC of the bladder is typically diagnosed in patients of advanced age, and it is usually muscle‐invasive and frequently metastatic at the time of diagnosis. Oncological outcomes remain poor, with the presence of metastasis and depth of bladder invasion serving as strong prognostic factors for survival. Treatment options include bladder‐directed therapies (transurethral resection of the bladder tumor, partial cystectomy, and radical cystectomy), radiotherapy, and chemotherapy. Multimodal therapy appears to offer benefits over single‐modality treatment. LCNECs of the urinary tract metastasize to the lymph nodes, liver, lung, brain, bone, adrenal gland, skin, and bowel [3]. Platinum‐based regimens are the initial treatment for metastatic neuroendocrine carcinoma of the bladder, and regimens containing cisplatin or carboplatin and etoposide are also common [4]. Taxane‐based anticancer drugs and immune checkpoint inhibitors are second‐line therapies [4].

In the present case, a solitary distant metastasis was identified twice. A maximum of three metastatic sites, all resectable or amenable to stereotactic therapy, is the proposed definition of oligometastatic bladder cancer [5]. The present case met that definition. While studies on metastasectomy differ in terms of patient characteristics and treatment approaches, metastasectomy may improve survival in highly select patients with oligometastatic bladder cancer [6]. For these reasons, metastasectomy was chosen in the present case.

Regarding metastasis‐directed therapy for neuroendocrine carcinoma of the bladder, surgical resection of a single metastasis and radiofrequency ablation for a single liver metastasis have been reported [7, 8, 9, 10]. Regarding patients treated with metastasectomy alone, some studies reported metastasis within 6 months [7], while others reported several years without metastasis [8]. Because the borders between brain metastases and normal brain tissue are unclear, complete surgical resection is difficult; therefore, radiation therapy is also used [9]. When radiation therapy and chemotherapy are used in addition to surgery, some patients do not progress [10] while others develop early metastases [9]. LCNEC of the bladder is highly malignant, and metastasectomy alone may be insufficient even if oligometastatic. To our knowledge, no reports have indicated whether metastasis‐directed therapy alone or with radiation or chemotherapy is preferable.

The present case was rare in that a single postoperative metastasis was observed twice, and long‐term survival was observed with only metastasectomy. One reason for the long‐term survival may be that neoadjuvant chemotherapy and surgery achieved pT0 disease [11]; an additional factor is that our patient underwent partial cystectomy. Radical cystectomy is a common procedure for large cell carcinoma of the bladder, but overall survival with this approach does not differ from that with bladder‐preserving surgery [12]. Another potential influence on long‐term survival may have been our patient's low‐risk Bajorin score. The Bajorin score is used to assess patients with metastatic disease and correlates with overall survival in metastatic large cell carcinoma of the bladder; it is based on Karnofsky performance status and the presence of visceral metastases [4]. However, there are reports that preoperative chemotherapy in small cell bladder cancer is used less frequently than previously reported [11] and that metastasectomy alone is insufficient [7]. If these improvements had been made, there might have been no second recurrence or even no recurrent metastasis.

The recommended follow‐up interval for patients with metastatic LCNEC of the bladder is 3–6 months [13, 14]; we also perform cystoscopy and CT every 3 months whenever possible because advanced and metastatic LCNEC of the bladder is associated with poor cancer‐specific survival [3]. Considering our experience with this patient, in the future, we plan to perform metastasectomy and adjuvant chemotherapy for single and resectable metastasis and chemotherapy for multiple metastases. Further cases are needed to assess the potential benefits of metastasectomy in neuroendocrine carcinoma of the bladder.

## 4 Conclusion

This case demonstrates that metastasectomy may be a therapeutic option for oligometastatic LCNEC of the bladder.

## Ethics Statement

The authors have nothing to report.

## Consent

Informed consent was obtained from the patient.

## Conflicts of Interest

The authors declare no conflicts of interest.
